# The Effects of Crystalline Admixture on the Self-Healing Performance and Mechanical Properties of Mortar with Internally Added Superabsorbent Polymer

**DOI:** 10.3390/ma16145052

**Published:** 2023-07-17

**Authors:** Guang-Zhu Zhang, Cen Liu, Xiang Ma, Xiao-Kun Yu

**Affiliations:** School of Civil Engineering and Transportation, Northeast Forestry University, Harbin 150040, China; zhangks@nefu.edu.cn (G.-Z.Z.); lc123@nefu.edu.cn (C.L.); mx520038@nefu.edu.cn (X.M.)

**Keywords:** crack, self-healing, crystalline admixture, superabsorbent polymer, mortar

## Abstract

Crystalline admixture (CA) can be incorporated into concrete to achieve self-healing of concrete cracks. In this study, both CA and superabsorbent polymer (SAP) were used as self-healing agents to investigate the effects of CA on the self-healing performance and mechanical properties of mortar with internally added SAP at different self-healing ages. The healing effect of cracks in mortar is assessed by crack observation and impermeability. The structure and composition of the filler in the cracks were analyzed by microscopic experiment. The experimental results indicate that CA enhances the healing of cracks in mortar specimens. The chemical reactions of CA primarily contribute to significantly improving the early-age crack-healing ability of the specimens, and the water absorption and expansion ability as well as the internal curing effect of SAP also facilitate the crack-healing process. Increasing the CA content leads to an increase in the Ca/Si ratio of C-S-H, causing a transition from a layered structure to a more compact needle-like structure. When 4% CA was added to the mortar, it resulted in an adequate formation of needle-like C-S-H structures, which eventually penetrate and fill the pits formed by SAP, compensating for the strength loss caused by SAP.

## 1. Introduction

Cement concrete is a widely acknowledged material that offers several advantageous features, including ease of construction, high strength, low cost, and high stiffness, which make it extensively utilized in the engineering domain [1]. However, due to factors such as temperature variations, loading, and non-uniform settlement, concrete is susceptible to cracking [2]. With an increase in service time, the ingress of aggressive ions such as chloride ions [3,4] and sulfate ions [5,6] from the environment can cause further damage to the interior of the concrete, resulting in reducing the impermeability and durability of the concrete structure [7]. The repair work of concrete cracks is crucial, and traditional repair techniques typically involve manual intervention as the primary means of repair. However, manual repair has certain limitations, such as the increasing cost with the deterioration of the crack condition [8]. Moreover, traditional manual repair techniques are inadequate in promptly and accurately detecting the appearance of cracks, especially when dealing with minor or remote ones, which can make the manual repair process even more challenging. Additionally, the repair procedure is both time-consuming and labor-intensive [9]. Self-healing technology for cement concrete enables cracks to self-repair without the need for manual intervention, resulting in reduced maintenance frequency, cost savings, and improved durability of concrete [10]. Thus, the self-healing technology of cement concrete has become an important topic of interest for scholars.

In the field of civil engineering, cement concrete self-healing technology can be broadly classified into two categories: one is autogenous healing, which involves the filling of cracks through the hydration of cement and the carbonation of Ca(OH)_2_ around the crack; however, this type of healing takes a long time and has limited efficacy [11]. The other type is autonomous healing, which relies mainly on the addition of special materials to concrete that can react and fill cracks [12]. The autonomous healing technology of cement concrete mainly includes microcapsule, microorganism, and crystal admixture self-healing technology [13]. Microcapsule self-healing technology refers to the encapsulation of healing agents, such as epoxy resin and cyclopentadiene dimer [14,15], within microcapsules, which are then mixed with a catalyst and introduced into concrete. When concrete cracks, microcapsules break around the cracks under stress and release healing agents. These healing agents react with the catalyst in the concrete and subsequently cure, thereby preventing further crack propagation and achieving the desired crack-healing effect [16]. In the study of Su et al. [17], it was reported that microcapsules possess a dense and sturdy structure, which enables them to withstand the shear forces generated during the concrete mixing process. However, Zuo et al. [18] have highlighted the limitations of microcapsule processing technology, which include complex processing procedures, a narrow range of particle size distribution, limited options for capsule wall materials, and the release rate of self-healing agents from the capsules, which also affects the effectiveness of the technology. Microbially induced calcium carbonate precipitation (MICP) refers to the introduction of microorganisms and essential nutrients into concrete to trigger microbial mineralization reactions, leading to the creation of calcium carbonate precipitates that can fill cracks in concrete and significantly improve its durability [19]. The advantages of MICP are its low energy consumption and minimal environmental impact. Unfortunately, Bayati et al. [20] stated that to achieve the desired effect of MICP technology, it is crucial to meet the requirements for microbial survival, control the temperature, and ensure the survival and reproduction of microorganisms in the highly alkaline environment of concrete (pH = 11–13) [21]. This means that only a limited number of microbial species are capable of meeting these requirements simultaneously. Consequently, these limitations may constrain the practical application of MICP technology in practical civil engineering.

CA is a type of waterproofing agent under hydrostatic pressure, composed of cement and sand containing reactive chemicals. Essentially, CA is a hydrophilic material that reacts readily with water and undergoes a chemical reaction with cement and water to produce an insoluble precipitate [22]. In recent years, some studies have confirmed that CA can generate insoluble precipitates through activation reactions, which can fill cracks in cement concrete. This process enhances the cement concrete crack-healing ability, thereby improving its durability and mechanical strength [23]. Zhang et al. [24] incorporated CA into an engineered cementitious composite (ECC) and investigated its impact on the mechanical properties and crack-healing ability of ECC by conducting experiments on compressive strength and crack observation. The experimental release that the compressive strength of the mortar, which contained CA, was 15.84% and 28.37% higher than that of the Control group after 28 and 56 days, respectively. These findings suggest that the addition of CA is advantageous in enhancing the compressive strength of concrete. Furthermore, experiments on crack observation have demonstrated that CA is more effective in healing cracks greater than 90 μm. The healing effect of concrete is significantly enhanced when the CA content in the mixture reaches 4.5%. Azarsa et al. [25] conducted chloride ion penetration tests and water permeability tests to analyze the influence of CA on the self-healing properties of ordinary Portland cement (OPC) and Portland limestone cement (PLC) specimens. The results verify that the addition of CA exhibits strong resistance to chloride ion penetration, and the mechanism may be related to the reduction in capillaries and porosity of the specimens after the addition of CA, as well as the microstructural characteristics of crystal deposition within a mortar matrix. During observations of crack healing, it was found that the CA specimens demonstrated good healing ability when the crack width was between 100–330 μm, and the healing rate reached 90% within the first 30 h, indicating that CA has a strong early crack-healing ability. It should be noted that the healing effect was better under water-curing conditions. Reddy et al. [26] set up four different curing conditions: water curing, wet–dry cycling, partial water curing (immersing 2 cm of the specimen in water), and dry curing to analyze the healing effect of CA. The results indicated that the compressive strength recovery rate of mortar with added CA reached 95% following crack healing under water-curing conditions. Microscopic experiments revealed that a substantial quantity of hydration products formed around the cracks of the CA-added specimens under water-curing conditions, suggesting that water presence significantly enhances the crack-healing effect of concrete with internal CA. Previous studies have shown that CA has excellent crack-healing abilities in the presence of water, enhancing the crack-healing rate by 7–10% compared to other experimental groups [27,28].

Previous studies have primarily focused on investigating the impact of external curing conditions on the crack-healing capacity of CA by altering the water content surrounding the specimen. However, limited research has been conducted to examine the influence of internal moisture variations in concrete on the CA’s crack-healing ability. SAP is a water-swellable high-molecular-weight polymer that exhibits strong adsorption and high water-absorbing capacity. By absorbing and releasing water, SAP can enhance the internal moisture conditions of cement specimens, thereby promoting internal curing effects and mitigating the self-shrinkage of cementitious materials [29,30]. In the study of Harn et al. [31], it was mentioned that SAP can promote cement hydration and fill cracks through their water absorption and expansion or internal curing, thus achieving the purpose of self-healing of concrete cracks. In a study conducted by Lefever et al. [32], nano-silicate cement was supplemented with SAP to investigate their crack-healing effect. The specimens were observed under a microscope, and the results showed that when the crack width was maintained at approximately 150 μm, the addition of SAP and nano-silicates resulted in the complete healing of 75% of the cracks. Deng et al. [33] investigated the impact of adding SAP to ECC for analyzing the crack-healing efficacy. The experimental outcomes demonstrated that SAP, when present in a moist environment, can expedite the self-healing process and enhance the mechanical recovery of ECC for cracks having a width of less than 50 μm via internal curing. In a dry environment, SAP promotes cement hydration by absorbing water from the adjacent areas with higher relative humidity and subsequently releasing it, thereby augmenting the self-healing capability of the cracks. Chindasiriphan et al. [34] investigated the healing behavior of cracks in mortar under the synergistic effect of rice husk ash and SAP, using a permeability recovery experiment. The results showed that the improved resistance to permeability of the specimens was partially attributed to the water-absorbing capacity of SAP, which transformed into an insoluble hydrogel, expanding instantaneously to seal the cracks. Additionally, it was found that SAP released the absorbed water to promote the hydration of cement in the mortar, and a mass of silicates was generated around the SAP, further enhancing the crack-healing effect. In this study, CA and SAP are chosen as chemical solutions to improve the self-healing effect of conventional cement. And CA has a chemical reaction, which reduces the permeability of mortar and promotes the self-healing of mortar cracks. In addition, SAP swells after water absorption to provide some contribution to mortar self-healing. The synergistic effect of CA and SAP contributes to the healing of concrete cracks, as well as enhancing the durability of concrete, ultimately extending the service life of concrete structures, and indirectly providing the possibility of environmental sustainability.

The purpose of this study was to investigate the effect of CA on the healing ability and mechanical properties of internally blended SAP mortar. To investigate the effect of CA and SAP on the mechanical properties of the mortar, compressive strength tests were conducted at 3, 7, and 28 days of standard curing. To analyze the effect of CA and SAP on the self-healing ability of mortar, the curing age of 3 days was selected as the zero point, and the load was applied to the specimen until the specimen produced stable microcracks. Then, the specimen was placed in water (20 ± 1 °C) for healing, and the observation was recorded by microscope at the healing age of 0, 7, 28, and 90 days. At the healing age of 28 days, the recovery state of the mechanical properties of the specimens after crack healing was assessed by the ratio of the compressive strength of undamaged specimens to that of damaged specimens. And the water absorption test was used to indirectly evaluate the healing effect of mortar by comparing the water absorption capacity of the healed mortar specimens. In order to further explore the self-healing mechanism of mortar cracks by CA and SAP, SEM-EDS and XRD tests were used to analyze the microscopic morphology and crystalline phase composition of the filler at the cracks. The innovations of this study are as follows: (1) SAP relies on the physical filling effect of water absorption and expansion to heal cracks within 7 days, whereas healing after 7 days relies on the internal curing effect to promote the generation of cement hydration products for crack filling. (2) The activation reaction of CA caused most of the cracks to finish healing within 7 days and obviously enhanced the healing ability of mortars with internal SAP. (3) The addition of 4% CA to the mortar results in an adequate formation of needle-like C-S-H structures, which eventually penetrate and fill the pits formed by the SAP, compensating for the strength loss caused by SAP.

## 2. Experimental Materials and Methods

### 2.1. Materials

The materials of the prepared mortar include ordinary Portland cement (OPC), ISO standard sand, SAP, CA, and superplasticizer. The particle size rate of SAP is 80–160 μm, and the main components are acrylamide and sodium acrylate, which were provided by Luban Building Waterproofing Materials Co., Ltd. (Guangzhou, China). Due to the high water-absorbing capacity of SAP, the SAP addition reduces the cement flow. Thus, to ensure unified fluidity, it is necessary to add a superplasticizer in the experimental group containing SAP. The CA used in this experiment is in solid powders. The chemical compositions of CA and OPC are shown in Table 1. It is worth noting that SAP is a highly absorbent polymer, mainly used in hygiene and convenience products, and in recent years, it has been applied in concrete as an internal curing agent and self-healing agent. SAP is more often used in concrete because of its inherent properties of high water absorption and swelling after water absorption, and it does not react with the hydration products in the cement matrix [35]. In addition, Tsampali et al. [36] have reported that the main components of CA are reactive silicate, finely ground blast furnace slag, and volcanic ash, parts of which are similar to the composition of cement. Thus, it is difficult for SAP to react with the constituents in CA.

The purpose of this study was to investigate the effect of the amount of CA on the self-healing properties and mechanical properties of mortar mixed with SAP. The mechanism of the synergistic effect of CA and SAP on the self-healing properties of mortar was revealed by compressive strength recovery, crack observation, a water absorption test, and microscopic tests. Because the crack-healing mechanism of CA alone for OPC was reported in previous studies, and the healing effect of CA on mortars with internal SAP has been less studied, the experimental groups of this study were selected as four groups, as shown in Table 2. The Control group, which only contained ordinary cement mortar, was named “Control”. The specimens in which SAP was added to the cement mortar to replace 0.5% of the mass fraction of cement were named “SAP”. The group in which SAP was added to replace 0.5% of the mass fraction of cement and CA was added at a mass fraction of 2% to the mortar was named “SCA2”. The group in which SAP was added to replace 0.5% of the mass fraction of cement and CA was added at a mass fraction of 4% to the mortar was named “SCA4”. The number of standard mortar specimens of 50 mm × 50 mm × 50 mm was twenty-six for each group, and the number of specimens used for each experiment is shown in Table 3. The water–binder ratio of all mixtures in this experiment is 0.5 (*w*/*b* = 0.5), and the sand–binder ratio is 2 (*s*/*b* = 2).

The mortar preparation process is as follows: (1) According to the mortar mix ratio, the standard sand, cement, CA, and SAP were put into the laboratory cement mortar mixing pot and mixed at low speed for 30 s. (2) Water containing superplasticizer was added to the cement mortar mixing pot and stirred at low speed for 2 min. (3) Stirring was paused for 30 s, the insufficiently mixed slurry attached to the inner wall of the mixing pot was scraped, and the mixer blade was scraped with a scraper blade. (4) Mixing continued a high speed for 2 min to obtain uniform cement mortar. After the preparation, the fresh mortar was poured into a 50 mm × 50 mm × 50 mm square mold. To minimize the bubbles in the cement mortar, the mold was placed on the cement mortar vibration table for vibration compaction. The surface of the mold was covered with a polyethylene film to prevent the loss of water in the cement mortar, and the specimen was left to solidify at room temperature (20 ± 1 °C) for 24 h before being demolded. Finally, the demolded specimens were placed in a standard curing room for curing (temperature 20 ± 1 °C, humidity ≥ 99%).

### 2.2. Methods

#### 2.2.1. Crack Prefabrication

**Figure 1:**
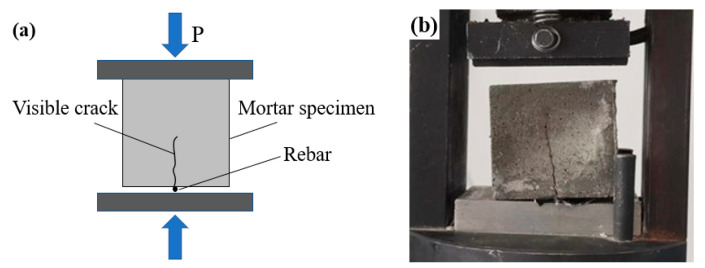
Crack preparation diagram: (**a**) schematic diagram and (**b**) device diagram.

Shrinkage cracks in concrete typically occur within a few days after casting and during the early stages of curing [37]. SAP can mitigate concrete shrinkage due to its water-absorbing properties at an early age [30]. Additionally, the self-healing of CA usually takes place at an early age after cement mixing. In a past study [38], Nasim et al. chose a curing age of 3 days as a “zero point” to study the effect of CA as a self-healing agent on the self-healing ability of specimens. Hence, to investigate the influence of CA and SAP on the early-stage crack-healing ability of mortar specimens, cracks were induced at a curing age of 3 days. The pre-cracking process refers to the application of load onto the specimen. According to the failure mechanism of concrete, stable microcracks with a width ranging from 0 to 1000 μm are formed when the pressure exerted on the specimen reaches 60% of its ultimate compressive strength [31]. When the specimen is subjected to loading, concentrated stress is induced at the contact point with the steel needle, leading to the occurrence of microcracks on the surface and inside the specimen as the load increases. A loading rate of 1 KN/s was confirmed according to ASTM-C109-21 [39]. Since the addition of CA can better heal cracks in the width range of less than 300 μm [40], in this experiment, cracks in the width range of 100–300 μm on microcracks were selected for observation. The crack preparation diagram is shown in Figure 1. The specific crack preparation steps are as follows: (1) Place a steel wire with a diameter of approximately 1 mm and a length of 50 mm at the center of the platen of the cement flexural-compressive testing machine. (2) Place the mortar specimen in the center of the platen, and apply the load to the specimen at a loading rate of 1 KN/s until a stable microcrack appears in the specimen. At this point, the value of the loading force is about 60% of the peak bearing capacity of the specimen after 3 days of curing, which is about 22 KN.

#### 2.2.2. Compressive Strength Test

Compressive strength experiments were used to test the impact of material on the strength of the mortar specimen. The experiment was conducted in accordance with ASTM-C109-21 [39] regulations. At the curing ages of 3 days, 7 days, and 28 days, compressive strength tests were performed on 50 mm × 50 mm × 50 mm cubic specimens using a DYB-300S fully automatic cement flexure and compression testing machine (Beijing Zhongjiao Jianyi Technology Development Co., Ltd., Beijing, China). Three specimens were tested for each group, and the experimental results were recorded to calculate the mean and standard deviation.

#### 2.2.3. Compressive Strength Recovery Test

The compressive strength recovery rate can indirectly reflect the healing effect of the crack. The pre-cracked mortar specimens were immersed in water for healing, and at the healing age of 28 days, the healed compressive strength was tested using a DYB-300S fully automatic cement compression and flexure testing machine. The compressive strength recovery rate ε is calculated according to Formula (1):

(1) $$\varepsilon =\frac{F_{1}}{F_{2}}\times 100\%$$

where ε—compressive strength recovery rate, %; $F_{1}$—compressive strength of the mortar at 28 days of healing age, MPa; $F_{2}$—compressive strength of mortar after 28 days of curing age, MPa.

#### 2.2.4. Microscopic Observation of Cracks

The closure of surface cracks on specimens is the most direct indication of their self-healing ability. Firstly, an Olympus SZ61 image-based 3D visual microscope (Olympus Corporation, Tokyo, Japan) was used to capture images of cracks on mortar specimens at a magnification of 2.5 times at marked locations during the healing ages of 0 days, 7 days, 28 days, and 90 days. Secondly, the photos were processed using Adobe Photoshop CS6 software, with the main steps including crack image binarization and outlier removal. Finally, for the processed output images, the histogram function of Adobe Photoshop was utilized to calculate the area of the white regions representing cracks and to determine the percentage of the white region area to the total image area. The definition of the crack-healing ratio η is given by Equation (2):

(2) $$\eta =\frac{A_{0}-A_{t}}{A_{0}}$$

where η—crack-healing ratio, %; $A_{0}$—the initial crack zone, ${mm}^{2}$; $A_{t}$—the crack area after t days of healing, ${mm}^{2}$.

#### 2.2.5. Water Absorption Experiment

**Figure 2:**
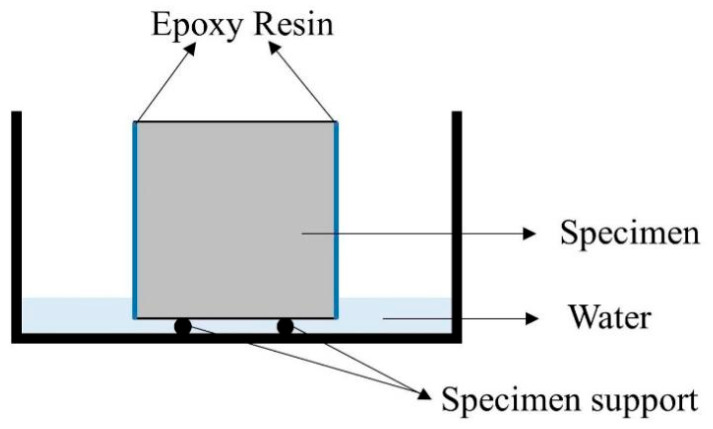
Water absorption test setup diagram.

The water absorption rate after crack healing reflects the healing condition of the crack. The experiment was conducted according to the ASTM C1585-20 [41] standard. The main operation process is as follows: First, at the 28 days of self-healing age, the specimens were placed in a drying oven at 50 ± 2 °C until mass variation was less than 0.1%. Second, apply epoxy resin was applied uniformly to all four sides of the specimen, except for the surface containing the crack and its opposite surface, to ensure one-sided water absorption. Third, the specimens’ crack faces were immersed in water to a depth of approximately 3 mm. The mass change in the specimens was recorded at 1 min, 5 min, 10 min, 20 min, 30 min, 45 min, 60 min, 90 min, 120 min, 180 min, 240 min, 300 min, and 360 min after immersion. The water absorption experimental device is shown in Figure 2. Finally, the water absorption height I (mm) is calculated using Formula (3), and the water absorption rate S (mm/t^1/2^) is obtained by calculating the slope of the I (mm) versus the square root of time (t^1/2^) curve, as shown in Formula (4):

(3) $$I=\frac{m_{t}}{A\rho }$$

(4) $$S=\frac{I}{\sqrt{t}}$$

where I—the water absorption height, mm; $m_{t}$—the change in mass of the specimen after t minutes, g; A—the area of the test surface of the specimen, ${mm}^{2}$; ρ—density of water, $g/{mm}^{3}$; S—the water absorption rate, $\left(mm/t^{1/2}\right)$.

#### 2.2.6. Microscopic Observation of Self-Healing Products

To investigate the composition of the filler at cracks of the specimen after healing, SEM was used for microscopic experiments. First, after a healing age of 28 days, samples were collected from the cracks of the specimens. The samples were broken into 3–5 mm small squares. Second, the broken samples were placed in a drying oven at 50 °C for two days until the weight change was less than 0.1%. Third, a magnetron sputtering coater was used to deposit a layer of platinum on the surface of the sample, and the imaging was then captured using SEM. To confirm the elemental composition of the fillers, the SEM-EDS technique was used to analyze the sample. In addition, this experiment further characterized the crystal phases of the filling material at the cracks through X-ray diffraction analysis (XRD). After 28 days of healing, copper was chosen as the X-ray target, with a 2θ range of 10–80° and a scanning step size of 0.02°. The samples were ground into powders and dried in a drying oven (50 °C) for two days. And the dried samples were taken out of the oven for XRD analysis. The obtained diffraction patterns of the samples were compared with the standard card in the database using MDI jade software to analyze the crystal phase composition.

## 3. Results and Discussion

### 3.1. Compressive Strength

**Figure 3:**
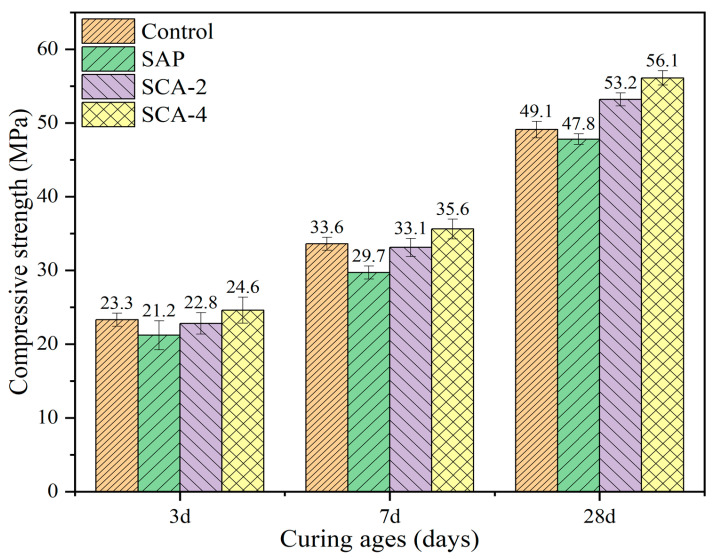
The compressive strength of the specimen at the curing ages of 3, 7, and 28 days.

Exploring the change of mechanical properties of specimens after adding materials is the first step in all engineering experiments. The compressive strength results are shown in Figure 3. The overall compressive strength of the mortar increases with the increase in the curing age. At the curing ages of 3 days and 7 days, the compressive strength of the SAP group decreased by 9.8% and 11.6%, respectively, compared to the Control group. This decrease can be attributed to the swelling of SAP particles, which creates the internal pores of the mortar specimen, resulting in a reduction in compressive strength. This phenomenon is consistent with that which was reported in Yang and Sun’s study [42,43]. The addition of SAP leads to a reduction in the compressive strength of the mortar, and the compressive strength of the SCA2 and SCA4 specimens is higher than that of the SAP specimens. Compared with the SAP group specimens, the SCA2 group specimens showed an increase in compressive strength of 7.5% and 11.4% at 3 days and 7 days, respectively, which is due to the activation material in CA reacting with cement to produce insoluble precipitation substances. This phenomenon was confirmed by SEM analysis, which showed that the gel substance was hydrated calcium silicate, filling the internal pores of the mortar specimens, ultimately leading to a denser mortar interior, resulting in an increased compressive strength. However, although the compressive strength of the SCA2 group increased with the addition of CA, it remained lower than the Control group. This suggests that though CA can enhance compressive strength, it is not sufficient to fully offset the negative impact of water absorption and expansion caused by SAP incorporation. The compressive strength of SCA4 at 3 days and 7 days reached 24.6 MPa and 35.6 MPa, respectively, which exceeded the Control group, demonstrating that the increase in CA dosage and sufficient CA content can increase the number of reaction products eventually generated. This increase in reaction products makes up for the strength loss caused by the addition of SAP, as confirmed by the SEM experiment, ultimately increased in the compressive strength of SCA4.

After curing for 28 days, the compressive strength of the SAP group was still lower than that of the Control group, with a value of 47.8 MPa. The compressive strength of SCA2 was 53.2 MPa, which was 8.3% higher than that of the Control group. The increased curing age promoted the hydration reaction to generate calcium hydroxide through the internal curing effect of SAP and promoted the activation reaction of CA to produce more insoluble precipitates, thus increasing the compressive strength. Compared with SCA2, SAC4 shows a 5.5% increase in compressive strength. This indicates that with the increase in CA content, the amount of activated reaction products of CA further increases, leading to a more compact internal structure of cement mortar and further enhancing the compressive strength of mortar specimens. 

### 3.2. Compressive Strength Recovery

**Figure 4:**
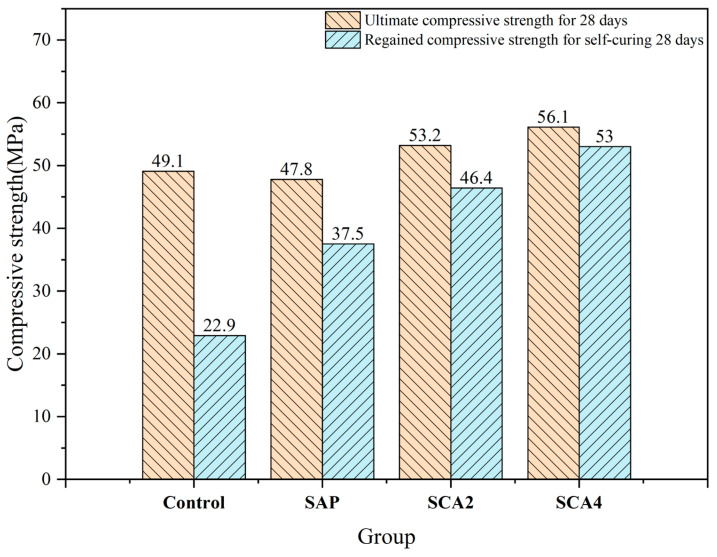
The ultimate compressive strength of the specimen after 28 days of curing and the compressive recovery strength after 28 days of crack healing.

The recovery rate of compressive strength includes indirect data reflecting the healing of the crack. Figure 4 illustrates the ultimate compressive strength of the specimen after 28 days of curing and the compressive recovery strength after 28 days of crack healing. At the healing age of 28 days, the compressive strength of the Control group was 22.9 MPa, and the recovery rate was 46.6%. Although the re-hydration products of unhydrated cement particles at the crack site can fill parts of the cracks, the ability of cement hydration to heal the cracks is limited, and the promoting effect of compressive strength recovery was not significant. The compressive strength of the SAP group was 37.5 MPa, and the recovery rate of compressive strength reached 78.5%. This result can be attributed to the water-absorbing capacity of the SAP particles, expanding into a gel-like state and filling the cracks present in the mortar specimen. In addition, the internal curing effect of SAP can promote further hydration of unhydrated cement particles at the crack, both of which contribute to the recovery of the compressive strength. The compressive strength recovery rate of the SCA2 group was 87.2%, which was further increased compared to the SAP group. This was because the added CA underwent an activation reaction to produce C-S-H, which made the mortar specimens denser. Moreover, a substantial presence of C-S-H filling at the crack site was observed under a SEM test, leading to a remarkable rise in the rate of compressive strength recovery in the SCA2 group. The compressive strength recovery rate of SCA4 was 94.5%, surpassing that of SCA2. This enhanced recovery can be attributed to the increased dosage of CA in SCA4, resulting in the production of C-S-H in both the mortar matrix and the crack. Furthermore, SEM experiments indicated that when a sufficient dosage of CA was used, the pits formed by the water absorption and swelling of SAP were almost filled with needle-shaped C-S-H crystals. As a result, SCA4 demonstrated the highest rate of compressive strength recovery.

### 3.3. Crack Observation

**Figure 5:**
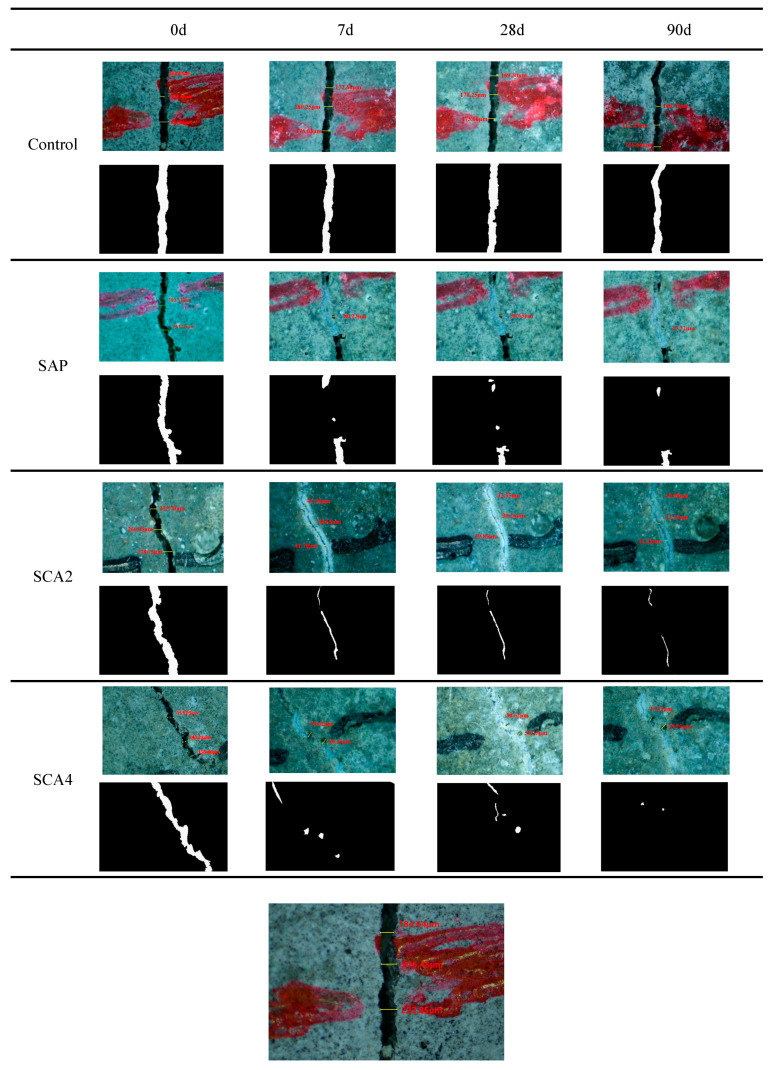
The healing effect on the surface of the cracks of the specimen at the healing ages of 0, 7, 28, and 90 days.

**Figure 6:**
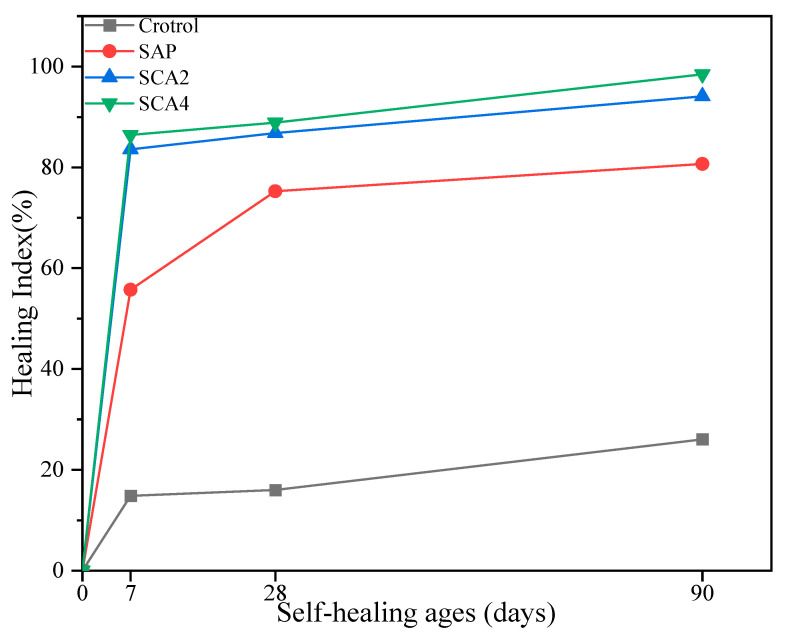
The crack-healing rates of the specimens at the healing ages of 0, 7, 28, and 90 days.

Microscopic observation at cracks can directly reflect the healing effect of cracks. Figure 5 shows the micrographs and processed images of the specimens at different ages, illustrating the crack surface healing effects. According to the results presented in Figure 5, it was observed that as the self-healing age increased, the Control group showed no significant crack healing, the SAP group showed partial healing, and in the SCA2 group, a noticeable white filler was observed at the crack location. When 4% CA was added, the crack was almost filled. Figure 6 is a plot of the crack-healing ratio versus time. As shown in Figure 6, at the healing age of 7 days, partial healing occurred in the Control group’s cracks. This is due to the hydration reaction of the unhydrated cement particles at the cracks in contact with the external water, which produces hydration products that fill part of the cracks. The healing rates of specimens with added SAP were 56.5%. In addition to the hydration reaction of cement, the added SAP has a high water absorption capacity. When cracks occur in the mortar specimens, SAP particles around the cracks absorb water and expand to form a gel, which fills the cracks and improves the healing rate. The healing rates of the SCA2 and SCA4 groups reached 83% and 86.6%, respectively. In addition to the SAP water absorption and the cement’s continuous hydration mentioned above, the addition of CA made the main contribution to the increase in the healing rate. The active substance in CA reacts with cement hydration products to generate C-S-H and fill the cracks, and the healing rate is significantly improved.

When the healing age was 28 days, the healing in the Control group was almost unchanged, indicating that the contribution of cement hydration to crack healing was not significant. The crack-healing rate of the SAP group increased to 75%. This conclusion is mainly attributed to the water-absorbing and swelling properties of SAP that fill the cracks. In addition, the internal curing effect of SAP [44] also contributes to crack healing. At a healing age of 28 days, SCA2 and SCA4 had healing rates of 87% and 89%, respectively. The increase in healing rate was due to the internal curing effect of SAP, which promoted the hydration reaction of the cement to produce more calcium hydroxide and activated the reaction of CA to generate insoluble precipitation filling cracks, thereby increasing the crack-healing rate. In Figure 6, it is noteworthy that the healing speed of specimens belonging to groups SCA2 and SCA4 exhibited a slowdown during the curing period of 7–28 days. This may be due to the activation reaction of CA consuming a considerable part of the cement hydration products after 7 days of healing. Consequently, there was an insufficient generation of products that could participate in the activation reaction of CA during the subsequent healing period. This led to a decrease in the healing speed of the crack. At the 90-day age, the healing rate of the Control group was only 20%, proving that the ability of cement hydration to heal cracks is very limited. The healing rate of the SAP group reached 80%, which indicates that the internal curing effect of SAP in the mortar continued. The enhancement in the crack-healing capability of mortar resulting from the secondary hydration of cement caused by the internal curing effect is highly effective. The healing rates of SCA2 and SCA4 reached 94% and 98.6%, respectively, at 90 days of healing, indicating that the specimens demonstrated outstanding healing effects under the combined influence of CA and SAP particles.

### 3.4. Water Absorption Rate

**Figure 7:**
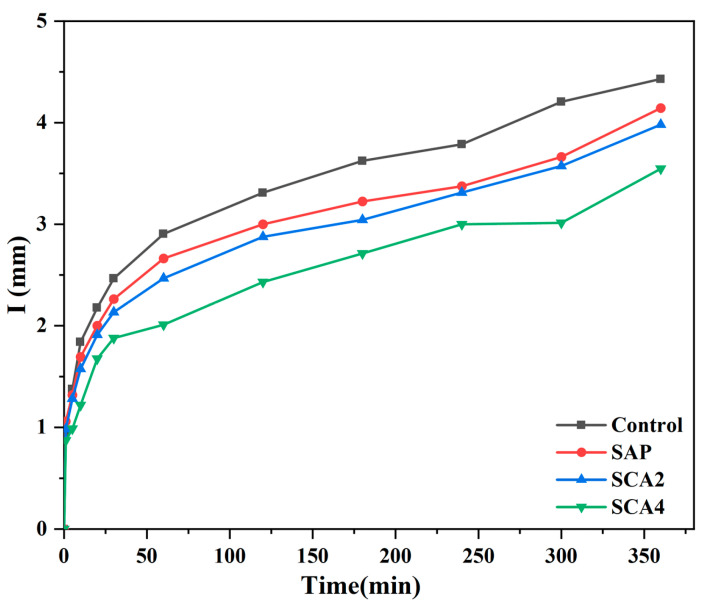
The water absorption rate of the specimen at the healing age of 28 days.

Water absorption can be used as an indirect means of assessing the healing status of concrete cracks. Alderete et al. [45] proposed that concrete cracking leads to an increase in water absorption, whereas the precipitates generated during the repair process can fill the cracks in the concrete, thus reducing its water absorption. The water absorption test results for the 28-day healed mortar specimens are shown in Figure 7, and the water absorption rate S (mm/t^1/2^) of the specimens calculated by Equation (4) is presented in Table 4. The order of decreasing water absorption rate based on experimental results was as follows: S_Control_ > S_SAP_ > S_SCA2_ > S_SCA4_. The maximum water absorption of the Control specimens was 0.057, which was attributed to the limited crack-healing capacity of cement hydration. After 28 days of curing, the crack-healing rate was only 20%, resulting in a relatively high water absorption rate. The water absorption rate of the SAP group was 0.053, lower than that of the Control. The reason for this phenomenon is mainly due to the fact that the SAP particles have absorbed water and expanded into a gel state, and parts of the cracks have been filled. Furthermore, the continuous internal curing impact of SAP promoted the hydration reaction of cement, generating Ca(OH)_2_ to fill parts of the cracks, reducing the water absorption rate even further. The SCA2 group’s water absorption rate was decreased by 0.003 compared to the SAP group. The further decrease in water absorption rate suggests that, in addition to the expansion and internal curing effects of SAP, the addition of CA is equally critical for crack healing. This is primarily due to the activation reaction of added CA, which produces C-S-H that fills the cracks. Additionally, the C-S-H produced by the activation reaction of CA makes the specimen interior denser, resulting in reduced water absorption of the specimen. The water absorption rate of SCA4 is 0.044, which was due to the increased amount of CA admixture, increasing the quantity of C-S-H produced. This promotes crack healing and densifies the interior matrix, resulting in the specimen’s lowest water absorption rate.

### 3.5. Microscopic Analysis

#### 3.5.1. Scanning Electron Microscope (SEM)

**Figure 8:**
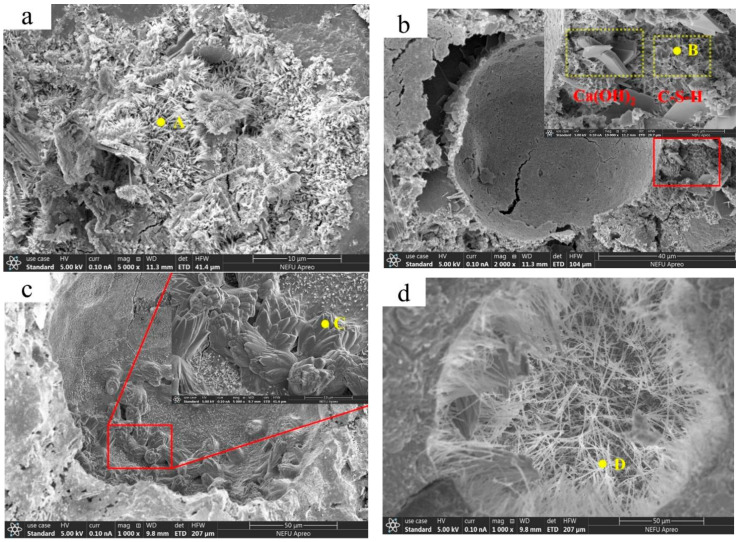
SEM images of the fillers at the crack of the specimens at the age of 28 days of healing: (**a**) Control, (**b**) SAP, (**c**) SCA2, and (**d**) SCA4.

SEM can observe the microscopic morphology of the reaction product of the healing agent. EDS was used to determine the elemental composition of the filler. The SEM experimental results are shown in Figure 8, and the EDS elemental analysis results are shown in Table 5. As observed in Figure 8a, the main filler at the cracks of the Control group were rod-shaped ettringite and disordered cluster-like hydrated calcium silicate substances, which were primarily hydration products from the cement hydration reaction [46]. The Ca/Si ratio of the C-S-H at point A was found to be 0.89 by EDS analysis. Figure 8b shows the microstructure of the SAP group, in which hemispherical pits can be observed. These pits mainly originated from the swelling of SAP particles upon water absorption. Furthermore, hexagonal plate-like Ca(OH)_2_ particles were found to disperse around the pits, which was attributed to the internal curing effect of SAP. As the cement hydration reaction proceeded, the water absorbed by SAP was released, promoting the hydration reaction of the unhydrated cement particles around SAP particles and producing a larger amount of Ca(OH)_2_ [47]. Additionally, Figure 8b reveals the presence of C-S-H with a similar morphology and structure as that observed in the Control specimens. The Ca/Si ratio of the C-S-H at point B was determined to be 0.87 through DES analysis. Figure 8c shows filler at the cracks of the SCA2 group. It can be observed that layered structures are distributed in the pits. Oliveira [48] reported that the layered structures are C-S-H. The Ca/Si ratio of the C-S-H at point C was found to be 1.16 through EDS analysis. In Figure 8d, a large amount of needle-like substance was observed distributed inside the pits formed by SAP in the SCA4 specimens. EDS analysis of point D showed a Ca/Si ratio of 1.3, confirming that the substance was C-S-H. SEM observations indicated that the addition of CA led to changes in the morphological characteristics of C-S-H compared to the Control group. Previous research has suggested that the Ca/Si ratio plays a crucial role in determining the morphology of the C-S-H structure [49]. The change in the value of Ca/Si is due to the introduction of Ca oxides and the active substance Si into the mortar with the increase in CA admixture, resulting in a change in the content of Ca and Si ions within the mortar, which eventually increases the value of Ca/Si. In the reports of Elsalamawy et al. [50], it is also mentioned that with an increase in Ca/Si, C-S-H eventually transforms into a needle-like structure. In addition, SCA2 and SCA4 specimens exhibit different morphologies of C-S-H, which is likely due to an insufficient CA dosage in the SCA2 specimens, resulting in a limited quantity of C-S-H formation and restricted growth of C-S-H within the internal space of the cement mortar, ultimately manifesting as a layered structure in the C-S-H. The SCA4 group, with the addition of 4% CA, generated a larger quantity of needle-like C-S-H structures, which ultimately penetrated the outer shell of the SAP, filling the pits formed by the SAP.

#### 3.5.2. X-ray Diffraction (XRD) Analysis

**Figure 9:**
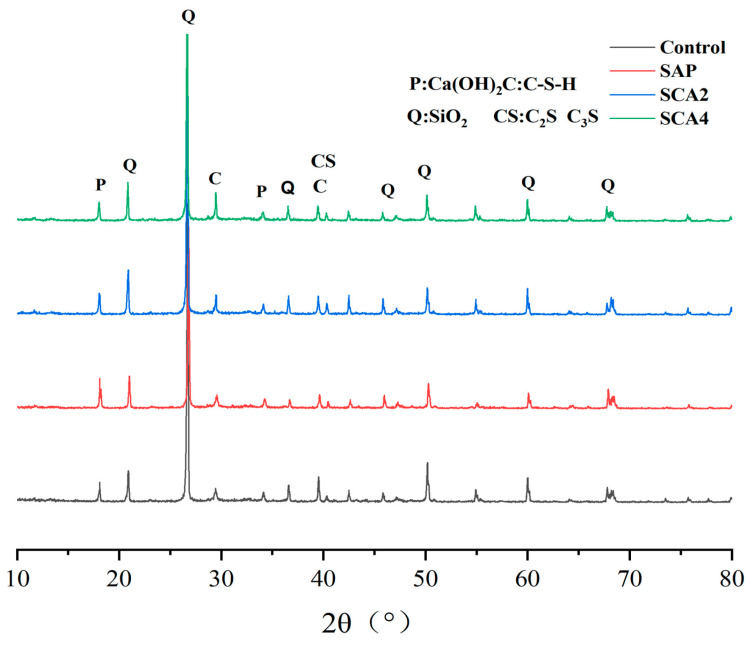
XRD pattern of the filler at the crack of the specimen after 28 days of healing.

The activating reaction of CA in a mortar and the internal curing effect of SAP in specimens will affect the composition of hydration products and ultimately impact the crack-healing performance. To investigate the crystal phase composition of the filler at the crack location, the fillings collected at 28 days after the healing of the specimen. They were well-ground and then analyzed by XRD. The analysis results are shown in Figure 9. The characteristic diffraction peaks of the filling material at the crack of CA and SAP mortar specimens mainly include Portlandite (PDF-04-0733) with 2θ values of 18.08° and 34.10° [51]; and C-S-H (PDF-33-0306) with a 2θ value of 29.35° [52]. In addition, Quartz with 2θ values of 20.86°, 26.66°, 36.47°, and 50.16° was also observed. Based on the SEM micro-experimental analysis, it was found that the filler at the crack location is mainly composed of C-S-H and Portlandite. Therefore, in this experiment, the variation in the diffraction peak intensity of Portlandite and C-S-H was semi-quantitatively analyzed. Compared with the Control group, the diffraction peak intensity of silicate at 18.08° increased due to the addition of SAP, which was attributed to the internal curing effect of SAP. The absorbed water released by SAP promoted the hydration reaction to generate more Portlandite, leading to an increase in the peak intensity. This phenomenon was consistent with the observed results in the SEM experiments. The peak intensity of Portlandite at 2θ = 18.08° decreased, and the peak intensity of C-S-H at 29.35° increased for SCA2 specimens, which is attributed to the addition of CA in the mortar. The activation of CA leads to the consumption of cement hydration product Ca(OH)_2_ and the formation of C-S-H. With the increase in CA content, the peak intensity of C-S-H diffraction in SCA4 increases significantly, which confirms that the increase in CA content can make the crack-healing effect more significant. Finally, the crack-healing rate of SCA4 is 98.6%.

## 4. Conclusions

This experiment investigates the effect of CA on the crack-healing ability and mechanical properties of SAP-incorporated mortar. The compressive strength test is adopted to analyze the variation in mortar strength after adding CA and SAP. The crack-healing effect in the early stage is observed and analyzed through experiments involving the compressive strength recovery rate, the visual crack-healing rate, and the water absorption rate. XRD and SEM-EDS were used to analyze the crystal phase composition and morphology of the self-healing products at the crack. The conclusions are as follows:

(1) The tests showed that although SAP had an adverse effect on the compressive strength of the specimens, the activation reaction of CA and the conservation effect within SAP could mitigate this adverse effect, and a higher dose of CA was more significant for this positive effect.

(2) The compressive strength recovery increased with the addition of CA due to the pits created by the addition of SAP, and cracks were filled with C-S-H. The product was formed by the CA activation reaction.

(3) The crack observation experiments showed that the combined action of CA and SAP could further improve the healing effect. The activation reaction of CA produced insoluble precipitation, which was one of the main reasons for the improvement in the healing rate of cracks. In addition, the water absorption and swelling of SAP and the internal curing effect also play a role in the filling of cracks. It is noteworthy that the crack-healing rate in the SCA2 and SCA4 groups slowed down from 7 to 28 days, because the CA activation reaction consumed more cement hydration products in the early stage, resulting in an insufficient amount of products involved in the reaction in the subsequent healing process.

(4) Water absorption experiments showed that the contribution to the reduction in water absorption because CA addition promoted crack healing and made the mortar matrix denser, much more so than that caused by swelling and the internal curing effect provided by SAP particles.

(5) SEM-EDS experiments showed that the morphology of C-S-H changed from a layered structure to a needle-like structure with an increase in CA, due to the increase in the Ca/Si ratio. In addition, when the amount of CA was insufficient, the restricted growth space led to the formation of a layered structure of C-S-H. Conversely, when the amount of CA is sufficient, a large number of needle-like C-S-H structures are generated, which eventually penetrate the SAP shell and fill the pits.

(6) XRD analysis showed that the addition of SAP promotes the generation of more Portlandite, which increases the intensity of the diffraction peak of Portlandite. Meanwhile, the CA activation reaction, which consumes Ca(OH)_2_, generates more C-S-H, resulting in an increase in the intensity of the C-S-H diffraction peak at 29.35°.

## Figures and Tables

**Table 1:** Chemical compositions of OPC and CA (wt.%).

Material		Composition (%)							
	CaO	SiO_2_	Al_2_O_3_	Fe_2_O_3_	SO_3_	MgO	K_2_O	Na_2_O	Loss on Ignition
OPC	65.84	19.84	4.74	3.33	3.12	1.24	0.88	0.15	0.86
CA	57.39	15.41	5.20	2.48	2.81	13.39	0.70	1.83	0.79

**Table 2:** Composition of mixtures.

Mixture	Binders (g)			Water (g)	*w*/*b*	Sand (g)	Superplasticizer (g)
	Cement	SAP	CA				
Control	1000	-	-	500	0.5	2000	-
SAP	995	5	-				0.7
SCA2	975	5	20				
SCA4	955	5	40				

**Table 3:** Numbers of specimens used for each group.

Experiment	Compressive Strength			Compressive Strength Recovery		Crack Observation				Water Absorption	SEM-EDS	XRD	Total Numbers
Curing age (days)	3	7	28	28	-	-				-	-	-	
Self-healing age (days)	-			-	28	3	7	28	90	28	28	28	
Control	3	3	3	3	3	4				3	2	2	26
SAP	3	3	3	3	3	4				3	2	2	26
SCA2	3	3	3	3	3	4				3	2	2	26
SCA4	3	3	3	3	3	4				3	2	2	26

**Table 4:** Capillary water absorption rate of all groups (within 30 min).

Group	Control	SAP	SCA2	SCA4
S (mm/t^1/2^)	0.057	0.053	0.050	0.044

**Table 5:** Calcium silicon ratio of different mixed groups.

Group	Control	SAP	SCA2	SCA4
Ca/Si	0.89	0.87	1.16	1.3

## Data Availability

Not applicable.
